# A case of 15q11‐q13 duplication syndrome and literature review

**DOI:** 10.1002/brb3.2219

**Published:** 2021-07-21

**Authors:** Zhuo Fu, Yue‐Xin Jia, Jun‐Xian Fu, Tian‐Xia Li, Jing‐Jing Zhao, Ting Wang, Zhi‐Dong Qiao, Xiao‐Yang Liu, Rong Tang, Ting Lv, Guang‐Lu Yang

**Affiliations:** ^1^ Department of Pediatric The Affiliated Hospital of Inner Mongolia Medical University Hohhot China

**Keywords:** 15q11‐q13 repetitive syndrome, autism spectrum disorder, chromosomal diseases, intractable epilepsy

## Abstract

**Background:**

The chromosomal 15q11‐q13 regions are structurally complex, and their abnormalities are associated with various neuropsychiatric disorders, including autism spectrum disorder (ASD), epilepsy, Angelman syndrome, and Prader–Willi syndrome.

**Case description:**

A 6‐year‐old child was admitted to the hospital as a result of an “epileptic status” showing ASD, intractable epilepsy, and total developmental retardation. Chromosome gene detection showed repetitive variation in the 15q11‐q13 regions, and the video electroencephalogram was abnormal. Although children are currently given antiepileptic treatment and rehabilitation training, intermittent seizures can still occur.

**Conclusion:**

The clinical phenotypes of 15q11‐q13 repetitive syndrome are complex, and vary in severity. Children with intractable epilepsy, ASD, and language and motor retardation should be considered to have this syndrome, which requires confirmation by multiplex ligation‐dependent probe amplification and gene detection. These approaches can enable early rehabilitation treatment and improve the patients’ quality of life.

## Graphical abstract

The chromosomal 15q11–q13 regions are structurally complex. Their abnormalities are associated with various neuropsychiatric disorders, such as autism spectrum disorder (ASD), epilepsy, Angelman syndrome, and Prader–Willi syndrome. A six‐year‐old child was admitted to the hospital as a result of having “paroxysmal seizures for 3 years and aggravated for 50 minutes,” showing ASD, intractable epilepsy, and total developmental retardation. Chromosome gene detection showed repetitive variation in the 15q11–q13 regions. Video electroencephalogram was abnormal. Although children are currently given antiepileptic treatment and rehabilitation training, intermittent seizures can still occur.

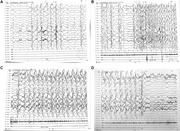

## 1 INTRODUCTION

Overall, 15q11‐q13 repetitive syndrome is an autosomal dominant genetic disorder characterized by different clinical manifestations based on the individual. The common manifestations include developmental retardation, hypotonia, bradykinesia, mental retardation, epilepsy, and autism spectrum disorder (ASD). Autism spectrum disorder is a complex neurological disease characterized by social difficulties, language disorders, and repetitive and stereotypical behaviors. Additionally, over 70% of children have mental retardation. Genetic factors are important causes of autism, and include genetic mutations and chromosomal copy number variants (Bill & Geschwind, 2009). Epilepsy is a common chronic nervous system disease that affects millions of people worldwide and often occurs in patients with chromosomal variants as well. Moreover, most patients with chromosomal variants develop language and motor retardation (Horsthemke & Wagstaff, 2008). Existing studies have shown that the 15q11‐q13 gene regions are closely linked to the occurrence of ASD. To improve understanding of the disease among doctors in all medical departments and reduce overlooked diagnoses and diagnostic errors, this paper reviews the case of a boy with 15q11‐q13 repetitive syndrome, accompanied by autism, epilepsy, and language/motor retardation, admitted to the Department of Pediatrics of the Affiliated Hospital of Inner Mongolia Medical University in July 2020.

## 2 CASE REPORT

A 6‐year‐old boy was admitted to the Affiliated Hospital of Inner Mongolia Medical University in July 2020 due to an “epileptic status.” At the age of three, he had developed convulsions during sleep with no obvious cause. The seizures manifested with loss of consciousness, head tilting back, eyes on the turn, cyanotic lips, foaming at the mouth, and stiff and shaking limbs. They were accompanied by urinary incontinence, and lasted approximately 4−5 min before self‐relieving, after which the child cried and screamed. No special treatment was effected at this time. The seizures occurred again after an interval of 2 months, with the same symptoms as before. After visiting a local hospital, the child was diagnosed with epilepsy. Oral administration of “lamotrigine 1/8 tablets, qd” was done, and the dosage was adjusted during the treatment. At the age of four, the patient had three seizures with the same presentation as before. He visited Beijing Children's Hospital in September 2019. The diagnosis was unknown, and rehabilitation treatment was recommended. At the age of six, about one convulsion per month was observed. The child visited a local hospital and received “sodium valproate 500 mg qd.” In May of the same year, he had five intermittent attacks. The boy stopped taking sodium valproate at home on May 29. At the end of May, he had another convulsion during sleep, presenting the same symptoms as before; the convulsion lasted for about 2 h, and then resolved. Another oral administration of “sodium valproate 500 mg qd” was given, and the patient had no attacks during treatment. However, he had another seizure during sleep at the age of 6 years and 7 months, with loss of consciousness, eyes on the turn, cyanosis of the lips, gurgling with phlegm, quadriplegia, and urine and feces incontinence. He was urgently admitted to our hospital. After oxygen inhalation and administration of chloral hydrate enema, the seizure was relieved, having lasted approximately 50 min. The child experienced dysphoria following the easing of symptoms.

### 2.1 Personal history

The boy was the first birth and first child of his mother; he was an easy delivery, but was overdue (45^+6^). His birth weight was 3000 g. There was no history of asphyxia or hypoxia following birth. No abnormalities were found during the neonatal period. He was able to sit by month 8 and walk by month 17. He could shout “Mom” and “Dad” at 3 years old. At the time of this study, he could speak complete sentences, but was unable to communicate normally with people.

### 2.2 Admission examination

No special examination is conducted.

### 2.3 Auxiliary examination

**FIGURE 1:**
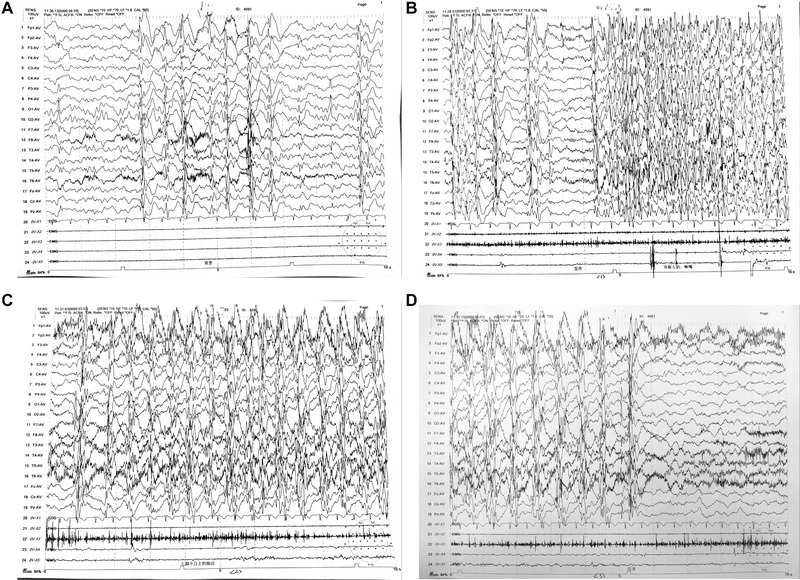
Video EEG: (A) Sleep period; (B) Wakefulness period; (C) Onset of ictal period; (D) Ictal period

The blood, urine, and stool routines, myocardial enzyme level, liver and kidney function, cardiac color ultrasound, abdominal color ultrasound, head magnetic resonance imaging, urine organic acid analysis, blood amino acid, and carnitine analysis were all normal. The video electroencephalogram (Figure 1) was abnormal for the child. Extensive/multifocal spike waves, slow spike waves, and multiple slow spike waves were emitted during each period. Sleeping discharge was in a nearly continuous state. Partial comprehensive secondary attacks were monitored in the right frontal pole and frontal region during the waking period.

### 2.4 Returned results of Pediatric Cardiac Scale (2016)

Major sports: intelligence age of subscale, 66; developmental quotient/index of subscale, 80. Fine movements: intelligence age of subscale, 31.5; developmental quotient/index of subscale, 38. Adaptability: intelligence age of subscale, 36; developmental quotient/index of subscale, 43. Language: intelligence age of subscale, 37.5; developmental quotient/index of subscale, 45. Social behaviors: intelligence age of subscale, 33; developmental quotient/index of subscale, 40. Intelligence age: 40.8. Developmental quotient: 49. Autism Behavior Checklist/Autism Scale Assessment Report: 56 points.

The above information was used in the clinical diagnosis of ASD. To date, the child has received oral administration of oxcarbazepine and lamotrigine, and has occasionally had seizures. Seizure frequency has been significantly reduced, and rehabilitation training has been carried out.

**FIGURE 2:**
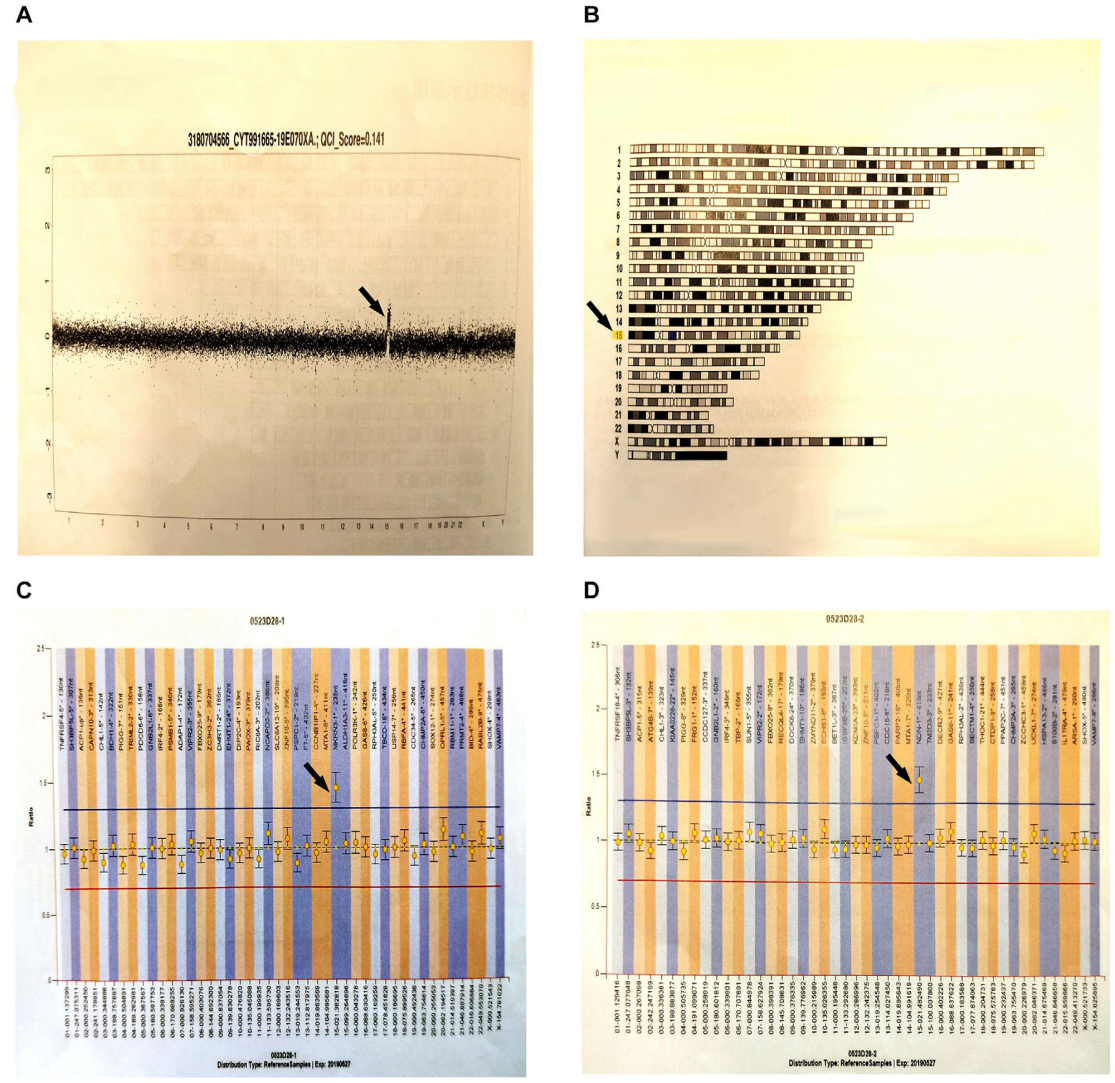
Gene test results. (A) A whole chromosome diagram: This diagram is the result of analysis. The transverse axis represents chromosomes 1–22 and sex chromosomes, and the longitudinal axis represents the signal ratio of tested sample to reference chromosome (expressed in log2ratio). The length of black segment and values represent the size of each segment and the mean of signals. (B) Chromosome karyotype diagram: The missing (loss) or amplified (gain) regions of analysis results are marked on the relative position of chromosomes. Deletion will be marked in red blocks and amplification in blue blocks. (C) After detecting MLPA P036, the exon1 repeat variation of MKRN3 is found at the 15q‐cen site in the subject. (The detection of subtelomeres involve two kits, namely P070 and P036. Test results need to be combined with other findings. Only if the exon on the same chromosome of the two probes mutates, the correct conclusion will be obtained.) (D) After detecting MLPA070, the exon1 repeat variation of NDN is found at the 15q‐cen site in the subject. (The results of both c and d indicate that the results of chromosome 15 are abnormal. Generally, the rearrangement of subtelomeres is existed in this site.)

After examination by medical ethics and family members using Agilent SurePrint G3 Human Genome CGH Microarray (array CGH) 8 × 60 K chip (Agilent Technologies, America) comparative genomic hybridization, follow the instructions. Luciferin (Cy3/Cy5) was applied to label the DNA of the patient and the reference samples, and then, microarray hybridization and post‐hybridization washing were performed. Fluorescence images were obtained using an Agilent chip scanner, and the data were read by Feature Extraction Technologies; standardized processing was performed. Data analysis was carried out using Genomic Workbench (Agilent Technologies), and regions of copy number change were found. Their position in the genome was determined based on the Human Genome Reference Sequence (GRCH37/HG19), available in the UCSC database since February 2009. Data analysis was performed using Gene‐related clinical information via Online Mendelian Inheritance in Man (OMIM: 608636). The test results showed arr 15q11.2‐q13.1 (23,769,039−28,527,183) × 3, 23,769,039−28,527,183, with a size of variation of 4.758 Mb (Figure 2). Amplified abnormalities of 1 were detected in 378 disease‐specific regions, and 41 subtelomere regions of the chip design: 15q11.2‐q13.1, according to published clinical literature, correlates with 15q11‐q13 repetitive syndrome. The genomic DNA of the blood samples was extracted using multiple linking probe amplification (MLPA) using a Qiagene Kit, sample quality inspection, DNA denaturation, ligation, gene amplification, computer application, and the genomic variation database. The test results showed MLPA 070. Repeated variation of exon1 of NDN was found at the site of 15q‐cen in the subject's chromosomes. The test results showed MLPA P036. Repeated variation of exon1 of the MKRN3 gene was found at the site of 15q‐cen in the subject's chromosomes; no large fragment variation was found at P106. The parents did not have the above variants. Considering the new variants, the child was diagnosed with 15q11‐q13 repetitive syndrome.

## 3 DISCUSSION

A large number of low copy repeats (LCRs) exist in the 15q11‐q13 regions, which are target areas for chromosome rearrangement, that in turn is likely to cause chromosomal fragment deletion and duplication, as well as extra marker chromosomes (structural maintenance of chromosomes) (Wang et al., 2018). There are five breakpoints (BP1–BP5) in these regions, where BP1–BP3 region deletion is associated with Prader–Willi and Angelman syndrome, and is influenced by parental origin. The copy number repetition in these regions is associated with 15q11–q13 repetitive syndrome (Horsthemke & Wagstaff, 2008; Urraca et al., 2013). Here, using the retrieval database established by the China National Knowledge Infrastructure, Wanfang data, and an analysis of the new retrieval functions in PubMed in September 2020, 25 cases with complete data reports were collected (locally and internationally). Combined with the case included in this paper, there were 26 patients in total. Among them, all 26 suffered from language retardation, 23 exhibited motor retardation, 23 had cognitive impairment, 20 had ASD, 16 had hypotonia, 10 exhibited special facial features, 9 had epilepsy, 6 had ASD, epilepsy, and language motor retardation, 2 had congenital heart disease, 1 had central precocious puberty, 2 had polyphagia and exhibited overeating, 1 had enuresis, and 1 died during follow‐up.

The child subject discussed about in this study, had 15q11‐q13 repetitive syndrome, which is characterized based on individual differences. He was treated in our hospital due to frequent seizures, which were followed by a post‐epileptic status; the treatment outcome was poor, even after prescribing various antiepileptic drugs. The child also suffered from autism, and language and motor retardation. There are two mechanisms of 15q11‐q13 repetition syndrome: the marker chromosome [inv dup(15) or idic(15)] caused by tetraploid duplication, and the intermediate duplication caused by trisomy duplication [int dup(15)]. The child had an intermediate duplication caused by trisomy duplication; this is a new variant, so clinical cases are relatively rare. Almost all int dup(15) cases recorded in the literature concern children with autism, who may also have retardation, mental disorders, and minor special faces; only a small number of children with epilepsy, the cases of children with language retardation, performance consistent with the literature of autism, but the children with no special features, and intractable epilepsy. Epilepsy is typically observed in patients with a chromosomal variation. Among the 25 other patients previously reported to have 15q11‐q13 repetitive syndrome, 29.2% had epilepsy. The severity of seizures in these cases was typically minor. Intractable epilepsy, ASD, hypotonia, and developmental retardation, were the manifestations of this syndrome. However, the clinical manifestations and severity differed with each child. Various genetic mechanisms have been proposed to explain the clinical heterogeneity, including the amount of chromosome replication, dose effects, and imprinting mechanisms of the genes in this region. The maternal genes in this genomic region may play a role in a dose‐dependent manner, and the copy number of these genes may be significant in terms of brain development. Alterations in epigenetic regulation lead to abnormal gene expression (Horsthemke & Wagstaff, 2008; Roberts et al., 2002). Hogart et al. analyzed the expression of different genes in 15q11‐q13 hexasomy and tetrasomy in male brain tissue. They believed that alterations in genetic copies, as well as additional effects linked to heredity or environment on epigenetic mechanisms, may affect the outcome and clinical heterogeneity of 15q11‐q13 repetitive syndrome (Hogart et al., 2009). Our case further demonstrated the phenotypic heterogeneity and clinical outcomes in this complex region.

Although epilepsy is a fairly common condition, not all patients with 15q11‐q13 repetitive syndrome exhibit it. In 2001, a study reviewed 15 patients with invisible 15q11‐q13 replication and found that 4 (26.5%) had epilepsy (Torrisi et al., 2001). One had minor epilepsy, one had temporal spinous wave type without clinical epileptic seizure during sleep, and three had occipital lobe epilepsy. All four patients were classified as having an unspecified type of epilepsy. The authors also reviewed 107 patients with an abnormal chromosome 15, among whom, 16 (43%) experienced seizures. Overall, they believed that the inconsistency of epilepsy manifesting in patients with 15q11‐q13 repetitive syndrome may be due to different repeat extensions, genetic backgrounds, or even exposure to different environmental factors. These findings were essentially the same as ours.

At present, there are few reports about 15q11‐q13 repetitive syndrome in the literature. Repeated locations and mutation types may have different clinical phenotypes. This study described a case of 15q11‐q13 repetitive syndrome, with intractable epilepsy, ASD, and language and motor retardation, and provided more references with which clinicians can identify the disease. Moreover, MLPA technology was used to detect gene rearrangement of chromosomal subtelomeres. Our patient had refractory epilepsy, developmental retardation, and ASD symptoms for unknown reasons, so we chose this technology, which can design a specific probe at the end of each chromosome, because it is economical, efficient, fast, and can reveal the etiology of unknown neurological diseases in some children. When clinicians encounter children with intractable epilepsy, ASD, language and motor retardation, or unusual facial features, they should consider 15q11‐q13 repetitive syndrome as a potential cause. This syndrome can be confirmed using the MLPA technique and gene diagnosis. Early rehabilitation training and treatment could improve the patients’ quality of life.

## CONFLICT OF INTEREST

The authors declare no conflict of interest.

## AUTHOR CONTRIBUTIONS

Zhuo Fu, Yue‐Xin Jia, Jun‐Xian Fu, Tian‐Xia Li, and Jing‐Jing Zhao participated in the clinical practice, including diagnosis, treatment, and consultation; Ting Wang, Zhi‐Dong Qiao, and Xiao‐Yang Liu contributed to the acquisition of data. Rong Tang and Ting Lv contributed to the analysis of data. Zhuo Fu, Yue‐Xin Jia, and Guang‐Lu Yang wrote the manuscript. All authors approved the final version of the manuscript.

## Data Availability

The datasets generated and/or analyzed during this study are not publicly available due to the lack of an online platform, but are available from the corresponding author on reasonable request.
